# The Relationship between Social Cohesion and Urban Green Space: An Avenue for Health Promotion

**DOI:** 10.3390/ijerph16030452

**Published:** 2019-02-04

**Authors:** Viniece Jennings, Omoshalewa Bamkole

**Affiliations:** 1 Southern Research Station, Integrating Human and Natural Systems, USDA Forest Service, 320 Green Street, Athens, GA 30602, USA; 2 Behavioral Sciences and Health Education, Rollins School of Public Health, Emory University, Atlanta, GA 30322, USA; omoshalewa.bamkole@alumni.emory.edu

**Keywords:** green space, social cohesion, public health, social capital, nature, ecosystem services

## Abstract

Social cohesion involves the interpersonal dynamics and sense of connection among people. Increased social cohesion can be associated with various physical and psychological health benefits. The presence of urban green spaces can encourage positive social interactions that cultivate social cohesion in ways that enhance health and well-being. Urban green spaces have also been linked to positive health behaviors and outcomes including increased physical activity and social engagement. Understanding the relationship between social cohesion and urban green space is important for informing holistic approaches to health. In this article, we discuss how positive interactions in urban green space can catalyze social cohesion, social capital and critical health-promoting behaviors that may enhance psychological health and well-being. We also summarize the strengths and limitations of previous studies and suggest directions for future research.

## 1. Introduction

The social environment plays an important role in the context of place, health, and well-being. Social cohesion, a key construct used to characterize the social environment, has been defined in many ways, yet it often refers to interpersonal dynamics and/or collective efforts that may be used to assess quality of life [1,2,3]. Social cohesion can also involve feelings of trust, belonging, acceptance [4], and connectedness [1,5] which often relate to positive social interactions. These favorable social constructs can encourage health benefits. For example, countries with high levels of social inclusion and cohesion tend to report favorable outlooks on their health at various levels of society [6]. Unfortunately, a blend of environmental and social stressors often make urban dwellers vulnerable to health challenges [7] such as those related to social isolation and limited time spent in nature. As more people spend the majority of time indoors and experience a “nature-deficit” [8], limited exposure to urban green spaces may also reduce opportunities for social engagement and the potential to develop social cohesion. 

Although prior research has shown that positive social interactions are associated with enhanced health [6,9], and exposure to green spaces may enhance health and well-being [10,11,12,13,14,15,16,17], few studies have directly explored the association between urban green spaces and social dimensions of health. Exploring how urban green spaces support social interactions and social cohesion can inform strategies to improve urban health [18]. In this article, we aim to bridge this knowledge gap by offering the first conceptual framework focused on social cohesion and urban green space. We also highlight key findings identified in the literature and conclude with recommendations to consider in future research. 

## 2. Urban Green Spaces and Aspects of Social Cohesion

Urban green spaces refer to areas such as gardens, parks, greenways, and other areas with grass, trees, and/or shrubs [19,20]. They can be common areas where people gather for leisure, social activities, and recreational purposes. Urban green spaces also afford opportunities for people to get outdoors and interact with nature and others in ways that may not occur in other settings. Reviews of the relationship between nature and health suggest that social cohesion is positively influenced by the presence and quality of urban green spaces such as parks and forests [4,10,21,22]. For example, a review on nature and health identifies social cohesion and increased social contacts as a major pathway through which the natural environment supports health promotion [4]. During a cross-sectional study in Western Australia, Francis et al. [23] found that the proximity to and the quality of parks were positively associated with sense of community. Thus, various activities and health promoting behaviors in urban green spaces may cultivate social cohesion and vice versa. In their study on urban parks, Peters et al. [24] analyzed activities (e.g., walking, cycling, having a barbecue, or a meeting) that may stimulate social interactions and social cohesion. While their observations varied by park location and sociodemographic variables (e.g., Dutch and non-Western migrants), urban parks were viewed as a place for social gatherings and other leisure activities [24]. Another study among visitors to neighborhood parks in New Orleans noticed that participation in park organizations can led to stronger perceptions of social cohesion [25]. Others also describe how strong social cohesion can encourage positive interactions that facilitate participation in clubs and organizations [26]. Thus, urban green spaces may support and potentially influence the social fabric of urban areas in a variety of ways. 

The following factors may relate urban green spaces to social interactions: an open park design to encourage active recreational activities [24], the availability of sidewalks [27], improved access to parks through quality transportation options [28], shaded areas that support relaxing environments [24], functional playgrounds [29], and the extent of organized activities [30]. Hence, characteristics of the built environment and amenities near urban green spaces maybe associated with social cohesion [31]. These studies also imply that the level of engagement within the green space (e.g., environmental stewardship and other volunteering) can vary based upon qualities of the green space (e.g., access and amenities), the intended use (e.g., for leisure and recreation), and an area’s overall social context. 

The ways that urban green spaces may support social cohesion prompts a need to broaden our perspective of their role in cities. Integrating the benefits of social cohesion with frameworks in the environmental and public health fields can inform how urban green spaces may enhance health and well-being. One example of this integration is the ecosystem services framework which describes nature’s benefits to human health and well-being [32]. Some suggest that social cohesion and sense of community are benefits from nature that align with cultural ecosystem services (e.g., aesthetic surroundings and outdoor recreation) [10,33,34] and are often underrated in health-related research [35,36]. By continuing to link the social benefits of urban green space to broader frameworks, we are better positioned to leverage our knowledge base and support interdisciplinary collaborations to improve individual, community, and societal health. 

## 3. Social Cohesion and Its Role in Public Health

Through the years, scholars have articulated the valuable role that social factors have upon human health and well-being [37,38,39,40]. These discussions also included social cohesion. Carpiano [5] presented a framework to illustrate how factors such as social cohesion relate to conditions, behaviors, and risk factors that ultimately influence one’s health. From a theoretical standpoint, the importance of social factors was expressed in Maslow’s Hierarchy of Needs, which identifies key components of psychological well-being such as belonging and sense of connection [41]. Maslow also noted that overlooking the importance of belonging can make our society more vulnerable to the demands of daily life and underestimate the value of a functional social environment [41]. However, transcendence (i.e., the ability to move beyond personal needs to communal benefits that support the common good) is included in later versions of Maslow’s hierarchy of needs [42]. The communal benefits of transcendence can also be linked to the shared values, collective efforts, and dynamics that support positive social cohesion. 

Some scholars frame the link between social cohesion and health through one of its subdomains—social capital [43]. Nieminen and colleagues used a multifaceted measurement of social capital to examine its relationship to self-rated health and psychological well-being. They observed a significant positive association between social capital (e.g., trust and social participation/networks) and psychological well-being; however, the relationship between social support and the outcome was attenuated (and no longer significant) when all dimensions of social capital were examined [44]. Across all age groups, the finding that individuals with high levels of social capital reported better psychological well-being compared to those with lower levels of social capital was consistent in their study [44]. In a systematic review on social capital and mental illness, De Silva et al. [45] analyzed twenty-one quantitative studies that primarily occurred in the United Kingdom or in the United States. Within these studies, sample populations were from all ages, including adults and children as young as two years old, and special populations such as individuals who were homeless, urban based individuals, and veterans [45]. Though the mechanisms are not defined, in each of the sample populations observed, the likelihood of mental illness (and negative consequences of mental illness) lessened with increased social capital [45] and social cohesion [46]. During their longitudinal study in Brazil, Secretti et al. [47] found that persons with lower levels of perceived social cohesion had a higher probability of developing a common mental disorder. Others note strong evidence to support that high levels of individual social capital correlate with fewer mental health challenges [45,48,49] and enhanced well-being [9,50]. However, the relationship between social capital and rates of common mental disorders are mixed and in need of further research [45]. Along with the aforementioned link to psychological wellness, social cohesion (or the lack thereof) can have implications for other health concerns. Previous studies suggest that aspects of social cohesion may reduce health challenges related to obesity [51], stroke [52], and cognitive decline [53] in some populations. For example, the degree to which people can depend upon their neighbor is associated with increased social cohesion and protection against negative health outcomes [9,46]. The presence of positive social cohesion can also support health related behaviors such as decreased smoking [54], less alcohol consumption [55], and increased use of preventative healthcare services [56]. Conversely, people who are socially isolated tend to be less healthy [57] and susceptible to stress [28], depression [58], and cardiovascular issues [59]. O’Doherty et al. observed that people with a low level of local trust and diminished social networks were more likely to report poor health [60]. Health stressors can also have disproportionate implications across socioeconomic groups [61,62,63,64,65], with those in urban and impoverished areas possibly more likely to experience negative health outcomes. Understanding the social dynamics within vulnerable populations can inform strategies to reduce health inequalities and disparities. Enhancing the social environment, particularly in disadvantaged areas can also support the pursuit sustainable communities [66] and health equity [67].

## 4. Potential Mechanisms and Pathways

As the context of place plays a powerful role in health and well-being, understanding the interplay between urban green space and social cohesion can inform strategic interventions to address health challenges. Generally speaking, social relationships can influence health through the following mechanisms: social engagement, social support (e.g., perceived and actual), social influence (e.g., developing norms), access to information, and increased contact with others [16,58,68]. These mechanisms can also be byproducts of social cohesion and social capital. As increased social contacts are a pathway to receive help with personal interests [58], high neighborhood cohesion can lead to more social organization and neighborly support (e.g., picking up mail and assistance with transportation) [9]. For example, social interactions in urban green spaces can provide opportunities to bond with others, develop their sense of community, and regroup from the demands of daily life [21]. Increased social contacts can cultivate a sense of community and other factors that inform our sense and perception of social cohesion [69]. To illustrate, gardens can provide a space for people to socially connect and grow nutritious foods [1,70,71], parks may support participation in athletic activities [72] as well as serve as a place for people to engage in other types of leisure, and urban forests can support outdoor recreation. Others suggest that the psychosocial processes related to positive social cohesion may be linked to greater support, increased self-esteem, and mutual respect between people [9]. 

Understanding the social environment of urban green spaces can support settings to increase physical activity and other healthy behaviors [73,74,75,76]. Some researchers suggest that areas where people feel safe and comfortable to walk are conducive to positive perceptions of social cohesion and promotes interest in using urban green spaces [77]. For example, a review article on parental factors involved in outdoor play found that a parent’s perceived level of neighborhood social cohesion is positively correlated with a child’s amount of outdoor play [78]. Similar studies describe how sense of community relate to walkability and quality built environment [79]. Hence, positive health behaviors such as increased physical activity may be related to the presence of or increase in social cohesion [80,81] and have implications for various health outcomes. A study in Australia found that social cohesion and recreational walking can partially explain the observed relationship between green spaces and mental health [82]. Also, people who reside in neighborhoods with greater social cohesion are more likely to know their proximity to the closest park and be aware of other resources to increase their physical activity [83]. Along with the positive association between use of green space and physical activity, social cohesion is often negatively associated with levels of stress [51,81,84]. This underscores the potential for well-designed urban green spaces to enhance the social environment by supporting an increase in social capital, more visitors to green spaces, and greater physical activity [74]. Thus, understanding the role of urban green spaces upon the social environment can support interventions for health concerns such as obesity [74] and psychological health challenges. 

**Figure 1:**
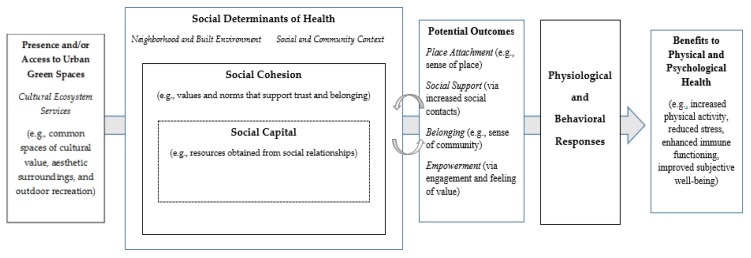
Conceptual framework to illustrate the relationship between cultural ecosystem services from urban green spaces and social cohesion (as a social determinant of health) with social and health outcomes.

Given these points, it is important to not overlook the role of social connectedness as an element of social cohesion that is crucial to health, particularly as it relates to urban green spaces. For example, after adjusting for sociodemographic characteristics, Maas and colleagues [85] analyzed the social mechanisms between green space and health in the Netherlands and found that residents with less access to green space had a greater perception of loneliness and limited social support. Figure 1 provides a conceptual framework to illustrate the relationship between cultural ecosystem services from urban green spaces and social cohesion with related social and health outcomes.

Our conceptual framework uses social determinants of health as an overarching domain which includes social cohesion and social capital. Social determinants refer to the conditions where people live, work, learn and play [86]. Social cohesion and social capital are distinct social determinants that can be linked to health promoting pathways [87,88,89] that can be facilitated through social contact with and within green space [10,90]. Accordingly, the presence and quality of urban green space may stimulate activities that contribute to increased social cohesion and various health benefits [91]. In a literature review, scholars illustrated how access to green spaces can facilitate positive social experiences which may be linked to social capital, sense of community, and empowerment, collectively influencing human health [92]. Some describe social capital as the interaction between people without a particular consideration of place, yet suggest that social capital can provide insight to understanding social cohesion [24]. Others differentiate the terms by characterizing social cohesion as the mutual values and norms that support favorable relationships and perceived belonging [4,5] while social capital pertains to the resources that one obtains through their social relationships [4,93]. Social capital can be further distinguished as bonding (e.g., resources accessed through a group or network) and bridging (e.g., enables one to access resources across their network) social capital [37]. Although the vague and varying definitions of social capital have garnered criticisms from academics, it still provides a useful context to describe a set of social dynamics that influence health and well-being [5]. During a cross-sectional study in the Netherlands, Cramm et al. [9] found neighborhood social capital and social cohesion were significantly associated with well-being in older adults. 

Our model also includes factors that we consider to be outcomes (or benefits) of increased social cohesion and social capital: place attachment, social support, belonging, and empowerment. We acknowledge that one could interpret the outcomes as precursors for social capital and social cohesion, hence the model has bidirectional arrows between these social domains and potential outcomes. One example is with place attachment which involves the bonds and meaning ascribed to a location [94]. The presence of urban green spaces may encourage the developed of place attachment. For example, during a social assessment of urban parks in Jamaica Bay (New York City), researchers found that urban parks foster social interactions, enhanced place attachment, and social resilience [95]. Access to urban green spaces can encourage place attachment along with sense of place and community satisfaction [10,24]. Others suggest that place attachment is linked with perceptions of fewer incivilities and local crime [96] in addition to increased subjective well-being [94,97] and other positive emotions [98]. This is relevant since sense of community may be linked to improved social support, psychological and physical health, less stress, and greater levels of physical activity [99].

Strong social relationships can be linked to stress-buffering benefits such as social support [100,101]. The opportunity to gather in urban green spaces such as parks can be valued by diverse members of society [102]. A study on interracial dynamics amongst urban gardeners in St. Louis found that they felt connected to their garden and perceived that community gardening gathered people from different backgrounds [103]. Similarly, Ward-Thompson et al. [28] examined the role of green space and the social environment on perceived stress among deprived urban communities in Scotland. They found that more urban green space coverage was linked with less stress along with the perception that green spaces (e.g., parks and open space) can encourage a sense of belonging and minimize social isolation in ways that mitigate stress [28]. As a sense of belonging involves feelings of acceptance and inclusion in social groups, it is also considered another mechanism between social ties and improved physical and psychological health [100,104]. For example, some perceive city parks as a way to connect with and strengthen ties with loved ones, neighbors, nature, and their community [105]. Thus, urban green spaces can potentially promote social cohesion through feelings of comfort, which connect people to particular places and with the people who visit them [24]. People who have a strong connection with nature often possess the capacity to experience heightened attention and mindfulness, which is linked to attention restoration [106]. A review article on potential mechanisms involved in the link between green space and health identifies enhanced immune functioning as a central pathway in this relationship [90]. Uchino [89] also provided insight from immune-mediated processes to suggest how social support can buffer changes in neuroendocrine, cardiovascular, and immune function [89]. However, more research on the biological mechanisms of green space and social cohesion is needed.

Putnam described how norms such as trust and mutual respect support mechanisms of social capital and contribute to broader forms of civic engagement [107]. As social cohesion includes aspects of trust and connection, it may also relate to empowerment and civic activities that can support positive change [108]. A study on parks in Portland, Oregon found that environmental stewardship and civic engagement provide opportunities for volunteers to interact and work toward a common goal that can foster community pride [109]. For example, some residents consider the subdomains of social cohesion (e.g., volunteerism, social engagement, and shared identity) and the presence of green space as valuable assets to enhancing quality of life [110]. Previous studies discuss the potential for urban greening and other outdoor activities to enhance social conditions in ways that can lead to empowerment [67,92,111,112,113]. However, these observations can vary based on the impact and support provided from such activities. The sense of connection that people can develop when they visit urban green spaces is another potential pathway to mediate the relationship between nature and human health and well-being [105]. Since the pathways that connect social cohesion, social capital, and health can vary by geographic scale [37], attempts to account for the micro-macro processes involved in social cohesion, urban green space, and health can be challenging. Overall, these are factors to consider as we gradually bridge this knowledge gap. 

## 5. Design and Methodological Approaches

Though limited in number, many studies on social cohesion, social capital, and psychological well-being often utilize a cross-sectional study design [45], which can limit our ability to discern a causal relationship [44]. One of the few studies employing a short prospective design (three years), examined social cohesion using a scale that asked participants about their feelings of neighborhood safety, perceptions of support, and trust [46]. A recent literature review found that interviews of community residents are a common approach to gathering data [22]. For example, Cramm et al. [9] used a cross-sectional study design and described social cohesion as the interdependencies among neighbors through a survey. Similar studies measure social cohesion through residential surveys at the neighborhood level [5,91] or a questionnaire related to safety, the extent of local acquaintances, and the willingness of neighbors to offer help [84]. In another study, Broyles et al. [74] surveyed park users on social cohesion and inquired if people in the park generally get along, if can they be trusted, and if they were willing to assist others. Other surveys incorporated questions related to the level of loneliness, social support, and extent of social contacts [85]. As the definition of social cohesion and social capital vary, the strategies to characterize and explore this topic can be framed by the respective study. De Silva et al. [45] measured social capital as an ecological variable, with a variety of measures (e.g., general trust in others and per capita organization membership) and multiple aspects (e.g., structural and cognitive) of social capital. Social engagement activities (e.g., volunteering) was also identified as a common indicator of social cohesion [114]. Others encourage the use of natural and quasi-experimental studies given the challenge of performing randomized controlled trials [18].

While the lack of data can present challenges, accounting for more confounding factors (e.g., walkable areas and access to other amenities) can enhance the research implications. Efforts to control for confounding factors can only be partially executed due to the array of factors that may influence social relationships and health [57]. Some studies incorporate factors such as income, education level, age, gender, economic deprivation [46], and the presence of children in the home [84] since they may affect the extent of social involvement [115]. Cramm et al. [9] adjusted for other individual characteristics such marital status, home ownership, and length of residence (in years). While length of residence can strongly affect one’s extent of social ties [116], this observation can vary by availability of social options, personal preferences, and the strength of a respective support system. As these studies focus on the relationship of social contacts and health, the insights they provide can inform how we approach research on green space (urban or otherwise) and social cohesion. Scholars who analyze urban green space have considered other factors such as proximity to parks [74], time spent in green space [16] different levels of urban development [85], and pet ownership [117]. Continuing to understand the factors involved can further develop empirical studies on social cohesion, urban green spaces, and health outcomes. 

## 6. Limitations

Researchers acknowledge a range of limitations with studies on social cohesion, urban green spaces, and health. It can also be challenging to define who counts as a neighbor or community member and how geographical boundaries intersect with social relationships that occur outside of the residential area [118]. Some scholars argue that the most important aspect of social cohesion is that it should be measured at the community-level [118]. However, it is also important to explore social cohesion (and social capital) beyond the neighborhood level [119]. The multiple ways that these social dimensions are measured throughout the literature may present limitations [44]. For example, some studies measure social capital with one item while other studies use more complex measures making it challenging to compare their findings [44]. There does not appear to be a standard way to measure social capital (and social cohesion) [60] and scholars note different dimensions of it would be informative [120]. It is possible that there are various ways to measure social integration and mechanisms of support across social demographics (e.g., age, race, economic status) and cultures [100]. Thus, it is possible that the influence of green space on individual health outcomes can vary by one’s level of social integration and support along with demographic factors such as age, race/ethnicity, and economic status. One study [20] also observed that the experiences people have while on green space can be mediated through one’s perceived social position. 

Although urban green spaces can encourage interactions between diverse residents and other opportunities that promote social cohesion [24], racial and ethnic minorities are often underrepresented in health questionnaires [84], hence the implications are often not fully applied to potential health inequalities and disparities. Together, these factors may limit the interpretations of social cohesion and related concepts to more westernized and independent cultural contexts [100]. The influence of spatial and temporal variation in social networks may also present limitations to this research topic. 

## 7. Next Steps and Future Research

Urban green spaces can support social aspects of health that are essential to the way a neighborhood functions [27] and a city thrives. Although many studies support the notion that social cohesion and social capital are determinants of psychological well-being [44,120], more research is needed to fully understand the role of social cohesion and its link between green space and health [4,84]. Few studies explore the link between social cohesion and factors such as the different types [27,28] and the quality of green spaces. Although the benefits of green space are becoming increasingly known, more studies are needed that focus exclusively on urban green spaces or compare the benefits by level of urbanity. Additionally, Jennings et al. [10] called for the benefits of urban green space to be further integrated with factors within social determinants of health, such as social cohesion (and social capital). This holistic approach can advance our understanding of multiple frameworks as they relate to ecosystem services, public health, and social equity [67,121]. Such insight also supports the need to link green space conservation to a larger social mission [122] that supports inclusive and effective engagement. 

As the level of social cohesion can exhibit spatial and temporal variation [76], some scholars recommend the use of longitudinal study designs and attempts to understand the role of active and passive uses of green space [82]. Longitudinal studies, which include observational and experimental approaches, allow the opportunity to observe changes over time, help one to identify a sequence of events, and analyze the interaction between potential risk factors [123]. Such studies could document the variation and extent of influence of social cohesion between green space access and health promoting behaviors over time [91]. However, cross-sectional and other types of studies can also enhance our knowledge on this topic. Understanding the differences between the unit of aggregation and the subsequent influence on population health can also be informative [118,124]. Although other aspects of the built environment can influence the extent of social interactions and mental health [76,125]; this is an area that warrants future research. For example, factors within the living context such as traffic volume, safety, and related infrastructure can potentially moderate the relationship between urban green space and health [126]. However, not all activities and characteristics of urban green spaces positively influence social cohesion. For example, Hong et al. found that the aesthetic appeal of green spaces may be beneficial to social capital, however older adults may perceive some green spaces (e.g., street trees and parks) as a concern for pedestrian safety [127]. Therefore, perceptions of crime and parks that are not well-maintained can limit the positive relationship between green space and social capital [27]. 

We propose that future researchers explore considerations such as: (a) investigate the mechanisms of how green spaces support social cohesion overall and in vulnerable populations; (b) consider the role of urbanization, a potential mediator of the relationship between green space and social cohesion; and (c) analyze how programming (social, recreational, and others) on green spaces foster a sense of belonging and residential retention. This information can consider the role of urban green spaces in the pursuit of social justice as it relates to equitable access to green spaces and decision making [128,129,130]. We also acknowledge addressing potential consequences of urban greening (e.g., gentrification) as another aspect to consider so that residents have sustainable access to and experience the benefits of green space. As they further investigate these areas, researchers can also consider the range of subjective factors that individuals use to select their social groups within their methodological approach [58]. More systematic reviews on social cohesion and social capital in relation to green spaces are also needed. 

Others suggest that future studies should be designed as prospective or intervention studies to determine the impact of social contacts for those who are socially isolated, such as the elderly [44,131]. While the complexity of social factors can make them difficult to model [27], there is a need for new datasets on these variables [58], which can help develop strategies to measure social cohesion. The design of studies on urban green space and health can vary by the health outcome of interest [132]. As we continue to account for different externalities associated with health interventions, additional insight can be obtained with greater knowledge on social determinants of health [58]. Although we mainly focus on the health benefits from the social cohesion and urban green spaces, it is important to acknowledge that aspects of social cohesion can have both positive and negative implications on health and well-being. For example, crime, incivilities, and social disorder are generally linked to detrimental health outcomes. Moreover, as neighborhood social cohesion may vary by socioeconomic status and in some cases be the product of exclusion or discriminatory practices, these occurrences may perpetuate gaps in health and social status [43]. This predicament can affect the type of meaning and attachment that different groups have to a particular green space [20]. Although the benefits from green space can relate to strategies in preventive medicine [121], researchers recommend that we investigate how health practitioners can encourage patients to become part of positive social groups and support their ability to identify with those in them. While we discuss findings focused on urban settings, additional research on social cohesion in suburban and rural settings can also be explored. This insight also supports the need to incorporate psychosocial factors in strategies in planning [105] and health promotion. We believe that our discussion on green spaces, social cohesion, and social capital will advance transdisciplinary research and inform programmatic strategies to promote nature-based health promotion.
